# Quarry Waste as Precursors in Geopolymers for Civil Engineering Applications: A Decade in Review

**DOI:** 10.3390/ma13143146

**Published:** 2020-07-15

**Authors:** Abbas Solouki, Giovanni Viscomi, Riccardo Lamperti, Piergiorgio Tataranni

**Affiliations:** 1 Department of Civil, Chemical, Environmental and Materials Engineering, University of Bologna, 40136 Bologna, Italy; piergiorg.tataranni2@unibo.it; 2 SAPABA, 40037 Pontecchio Marconi BO, Italy; g.viscomi@sapaba.it; 3 Steer Group, 40126 Bologna, Italy; riccardo.lamperti@steergroup.com

**Keywords:** quarry dust, mine tailings, aggregate recycling, alkali-activation, waste powder, calcination

## Abstract

Carbon footprint reduction of paving materials could be explored through recycling mining by-products into different applications, which will preserve natural resources and decrease environmental issues. One possible approach is to reuse quarry dust and mining ore waste as precursors in geopolymer applications. geopolymers are mineral polymers rich in aluminosilicates with an amorphous to a semi-crystalline three-dimensional structure. The current review aims to summarize the studies conducted during the past decade on geopolymers containing quarry dust and mine tailings. The first section discusses various precursors used for geopolymer cement production such as metakaolin, ground granulated blast furnace slag (GGBFS), fly ash, and quarry/mining ore wastes including silt, tungsten, vanadium, copper, gold, zinc, marble, iron, basalt, and lithium. Different calcination treatments and curing conditions have been summarized. In some cases, the precursors are required to be calcined to increase their reactivity. Both ambient temperature and elevated temperature curing conditions have been summarized. Less attention has been paid to room temperature curing, which is necessary for field and industrial implementations. Engineering properties such as compressive strength, density, durability and acid resistance, water absorption and abrasion of geopolymers containing mining waste were reviewed. One of the main barriers preventing the widespread use of waste powders, in addition to economic aspects, in geopolymers could be due to their unstable chemical structure. This was shown through extensive leachate of Na^+^ or K^+^ cations in geopolymer structures. The review of over 100 articles indicated the need for further research on different aspects of quarry waste geopolymer productions before its full industrial implementation.

## 1. Introduction

Road and construction sectors are practicing greener solutions where aggregate recycling has become an essential principle for most authorities. Various studies have investigated the feasibility of recycling 100% of asphalt pavements [1,2,3] or have tried to produce concrete using construction and demolition waste [4]. Nevertheless, aggregate quarries still must keep up with the global demand for virgin aggregates. The aggregates are transported to the processing plants where they are first crushed into smaller and manageable sizes. The crushing and milling stages are usually followed by screening and washing of aggregates prior to final stockpiling. The water used during the washing process is directed to precipitation tanks for further processing. The resulting residue, which is mainly consisted of dirt and very fine powdery substances, are piped out into artificial lakes and ponds called tailing mines. These materials, mostly mineral fillers from plant processes, could become an environmental issue because of the landfilling limitations and strict legislation on their disposal.

Over the past decades, various techniques and methods have been employed as solutions for recycling mining waste. For instance, tungsten mining waste has been processed and used as a filler in asphalt pavements. The study substituted the mining waste with the typical limestone filler in asphalt pavement. The results did not indicate any adverse effects of the waste filler on the performance of tested hot mix asphalts [5]. With a similar concept, magnetite was used as a filler in various mastic mixtures with the aim of providing a solution for recycling mining waste and microwave healing of asphalt pavements [6]. Other approaches to recycling waste mineral fillers include the application of mining waste in cement paste backfill [7], recovery of valuable metals [8,9], paint production [10], carbon capture and storage [11], and concrete production [12]. All methods follow one aim, which is to reduce the impact of mineral waste on the environment [13].

Alternatively, some quarry wastes are both rich in aluminosilicates and have the proper mineralogy, which allows their application as precursors to produce geopolymer cement and similar synthetic materials. Geopolymers are alternative binders which have diverse applications in various fields such as coatings and adhesives [14], fiber composite production [15], decorative stone artifacts [16], thermal insulations [17], building materials, low-energy ceramic tiles [18], waste encapsulation [9], thermal shock refractories [16], biotechnologies [19], etc. Geopolymers were first introduced by Joseph Davidovits in the early 1970s, where his primary aim was producing nonflammable and noncombustible plastics. During his studies he discovered that the synthesis of some organic plastics in alkali solutions as well as mineral zeolites and feldspathoids were driven by the same hydrothermal conditions. Through further reviewing of zeolites synthesis-related patents and literature he realized a gap, which had not been investigated before. Afterwards, he developed materials rich in aluminosilicates with a three-dimensional amorphous to a semi-crystalline structure and coined it as geopolymers [16,20]. In other words, geopolymers are mineral polymers produced through geochemistry or geosynthesis and their discovery has increased the scientific interests and application of a new class of inorganic polymers over the past decades. For instance, kaolinitic clays were wet mud and were only processable through compression or extrusion. However, in 1975, a geopolymer in the form of a liquid binder was discovered and patented, which included metakaolin and soluble alkali silicates [16]. The mineral resin (metakaolin + Na-silicate + NaOH) had an exothermic reaction, which was advantageous since it helped the hardening of thick materials. In addition, the sodium silicate (SS) increased the hardening speed of the liquid binder.

Geopolymer cement and concrete have been widely used in different civil engineering application. They are produced with low processed natural materials or industrial by-products, minimizing carbon footprint. The presence of calcium cations mainly obtained from GGBFS provides room temperature hardening for geopolymer. In addition, the setting time of geopolymers are faster compared to normal concrete yet slow enough to allow plant to site transportations [21]. Geopolymers are not affected by common durability issues related with conventional concrete and could outperform conventional concrete in many aspects. For instance, the maximum reported compressive strength for geopolymer samples produced with loess and fly ash was reported as 113.8 MPa, which is much higher than those obtained by ordinary Portland cement (OPC) [22]. High performance Portland cement (HPC) was compared to geopolymer concrete in terms of mechanical and thermal behavior and microstructure properties [23]. Regarding compressive strength, geopolymer concrete showed faster setting times up to 15 MPa just after two hours. However, the strength of both mixtures became similar after 7 days of curing and maxed at about 60 MPa after 2 years. In terms of porosity and microcracks, geopolymer concrete had formed a denser structure compared to HPC. Lastly, thermal analyses by Differential scanning calorimetry (DSC) and Thermogravimetric analysis (TGA) indicated fire resistance of geopolymer concrete compared to HPC.

Geopolymer cement is categorized into four different types including slag, fly ash, ferro-sialate, and rock-based geopolymer cements [16,21]. Each type is composed of different materials, mix design, and consequently different curing/processing procedures. Regardless of the type, most systems use alkali solutions consisting of NaOH and soluble sodium silicates. The very first geopolymer containing slag was produced in the early 1980s. This type of geopolymer consisted of metakaolin, ground granulated blast furnace slag, and alkali silicate solution [21]. Fly ash geopolymers are mainly produced by two different methods. The first group contains fly ash and a very strong alkaline solution. This system requires heat curing of up to 48 h at temperatures ranging from 60 to 85 °C. On the other hand, geopolymers made with fly ash and blast furnace slag do not require heat curing and use a user-friendly alkaline solution [9,24,25]. Rock-based geopolymer cement allows the utilization of volcanic tuffs and mine tailings within the geopolymer system. These geopolymers have lower CO_2_ footprint and better properties compared to the normal slag-based geopolymers.

Mine waste management has become an important aspect during the past decades and various methods have been proposed for reducing its impacts on the environment. In this regard, geopolymer cement has become an interesting and alternative method for mine tailing management [5,26,27]. The number of published articles on geopolymers has increased dramatically. Thus, the aim of this paper is to review and summarize published data related to geopolymer cement and concrete containing waste mine tailings and quarry dust.

## 2. Geopolymer Composition

In general, a geopolymer consists of two essential parts including precursors and hardeners/activators. The precursors are raw materials rich in alumino-silicate oxides with high reactivity. The precursors are mixed with liquid hardeners to form a geopolymeric gel, which consequently hardens because of geopolymer condensation. Different precursors have been used in various research studies such as fly ash [22,28,29], metakaolin [30,31,32], and blast furnace slag [33,34,35,36]. In most cases, the reviewed studies have investigated the feasibility of using quarry dust, mine tailings, or industrial waste as precursors in geopolymer cements and binders [37,38,39,40,41,42,43,44,45,46]. Since quarry dust and mining by-products are the focus of the current paper, a separate section has been dedicated to them.

### 2.1. Precursors

#### 2.1.1. Metakaolin

Calcined kaolinitic clays otherwise known as metakaolin (MK) were one of the first precursors used in geopolymer research. The initial application of MK was mainly in paper and plastic industries where it was used as filler. However, about four decades ago, a flash calciner technology was used instead of a normal rotary kiln furnace to produce a new type of metakaolin named Argical^®^ [47]. Today, various chemical companies have produced different types of metakaolin, which are suitable for different applications. The composition of metakaolin is mainly made of SiO_2_ and Al_2_O_3_ with a small percentage of metal oxides. Davidovits (2019) investigated the exothermicity data of several metakaolin and indicated the minimum time required for each metakaolin to reach its highest thermal peak in an alkaline solution [47].

By reviewing various studies during the past decade, it becomes evident that metakaolin has kept its popularity as the main precursor among different researchers. However, in recent years, with the aim of recycling as much industrial waste as possible, the popularity of metakaolin has decreased. For example, recently in an attempt of reducing inorganic waste, up to 70% of tungsten waste, along with only 10% of metakaolin were used as precursors in an alkaline-activated foam mix design [30]. In addition, the authors used aluminum powder as the foaming agent and claimed that the strength of the final product was equal or even higher than samples made with only MK and fly ash. In a different approach, vanadium mine residues were used with metakaolin for geopolymer production [42]. Chemical and mineralogical studies indicated that part of the mining waste favorably reacted with the soluble silicon and the unreacted section acted as aggregates in the mixture.

#### 2.1.2. Fly Ash

Coal power stations are still one of the main solutions for providing electricity worldwide. At electricity power plants, finely powdered coal is injected with air into a combustion furnace where it immediately burns at very high temperatures. Consequently, the shales and clay inside the suspended coal that are rich in silica, aluminum, and iron melt. Upon rapid cooling, the material solidifies into spherical material commonly known as fly ash. Fly ash has been used as a precursor in geopolymer cement and binder production throughout the world. Based on plant type and combustion temperatures (ranging from 800 to 1800 °C), the final chemical composition of fly ash may differ (Table 1). Therefore, an X-ray diffraction (XRD) analysis could aid the selection of appropriate fly ash for geopolymer production. In this regard, the properties of appropriate fly ash for alkali-activation has been suggested [48,49]. The papers indicate that to ensure a proper alkalization, the fly ash should have a Si/Al ratio between 2 and 3.5. In addition, the percentage of Fe_2_O_3_ should be lower than 10% while the percentage of unburnt material should be as low as 5%. Furthermore, fly ash should have a high vitreous rate and the percentage of active silica such as glass should be above 40%. Regarding particle size, it has been suggested that between 80 and 90% of the particles should be smaller than 45 µm. The mentioned characteristics could improve the final geopolymer product. However, high calcium, quartz, and mullite content should be avoided. The reactivity of Si/Al in the fly ash would be affected by high quartz and mullite (>5%) content, whereas high CaO could initiate a fast setting of geopolymer paste during preparation [50].

**Figure 1:**
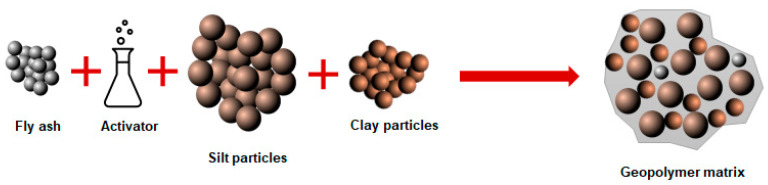
Loess stabilization using geopolymer binder [22].

Geopolymer cements and binders have various applications in building and construction projects. For instance, in an attempt to recycle quarry dust, geopolymer adhesive mortars were made using fly ash, crushed stone dust with different NaOH molarity and ratios [44]. Considering energy levels, waste and raw material usage, a maximum amount of 67% of quarry dust was proposed as the most efficient quantity during geopolymer production. Moreover, the authors suggested that fly ash and the quarry dust formed a homogenous mixture. In a separate attempt, the potential of recycling marble quarry dust into a geopolymer system was investigated [51]. In this regard, the geopolymer mixture consisted of fly ash, blast furnace slag, clay, gypsum, concrete, and marble sludge. The results indicated that the mixture containing no clay and gypsum had shown the highest compressive strength rate of 52 MPa. This is interesting since Davidovits [16] has claimed that the addition of gypsum inhibits the geopolymer chain reaction and may lead to adverse results. The applications of geopolymers are vast and are not only limited to mortars and cements. For example, in a study conducted by Yliniemi et al. (2017), gold, copper, and zinc tailings were incorporated into the fly ash geopolymer system to produce artificial aggregates. The aggregates were then used in mortars and concrete specimens, which showed enhanced mechanical properties compared to LECAs (light expanded clay aggregates) [52]. The application of fine materials into aggregate systems and unbound materials has been given negative feedback through the works conducted in the soil mechanics field, which usually result in reduced resilient modulus and increased permanent deformation. Therefore, in some cases, geopolymers have been employed as a greener solution for soil stabilization. Loess is formed from clay and silt particles, which provides for bearing capacity. However, the material will deteriorate and eventually collapse in case of direct water contact. An experimental study evaluated the possibility of loess stabilization through fly ash-based geopolymerization [22]. The authors claimed that the fly ash geopolymer binder bonds with the loess and provides a stable structure. A conceptual microstructure model regarding loess stabilization using geopolymer is shown in Figure 1.

#### 2.1.3. Ground Granulated Blast Furnace Slag

During iron manufacturing process fluxing agents are added to the molten iron to produce molten slag. The molten slag is immediately quenched, which initiates the granulating process. The granulated slag is then dried and ground to produce ground granulated blast furnace slag (GGBFS). The slag is rich in aluminum, magnesium, and calcium-silicates with a semi-crystalline glass-like structure. In general, the composition of slag consists of SiO_2_, Al_2_O3, CaO, MgO and between 44 to 47% of the total composition is made up of SiO_2_, +Al_2_O3 [16]. Slow cooling procedures such as air-cooling method would not produce hydraulic or pozzolanic phase. Consequently, air-cooled slags will not be appropriate for geopolymer production.

Various papers have included blast furnace slag as a precursor for producing geopolymers containing quarry waste [29,33,34,36,37,43,53,54,55,56,57,58,59]. The potential of using geopolymers consisting of copper mine tailings and slag for pavement construction was investigated [33]. The addition of 50% GGBFS increased the unconfined compressive strength (UCS) by 20 MPa compared to samples produced with 0% GGBFS. The increase in the UCS was related to large surface area and higher chemical reactivity, high smelting temperatures and the amorphous structure of the GGBFS. In addition, the water to solid ratio dropped by 15% with the addition of 50% GGBFS compared to the control sample. The reduction of the water content decreased the required NaOH and sodium silicate in the mixture, which could be a benefit in terms of production costs.

Ambient temperature curing for geopolymer mixtures containing GGBFS could be applicable because of the presence of calcium cations. In most scenarios, this would eliminate the need for elevated temperature curing and could facilitate industrial implementations. For instance, the addition of slag to alkali activated fly ash was investigated [60]. In addition to room temperature curing, two fly ash samples were also cured at 60 °C. The latter showed higher UCS compared to same samples cured at ambient temperature. However, the mixture containing 15% slag showed a maximum strength of 47.5 MPa, which was very similar to the USC of samples merely made with fly ash and cured at 60 °C. Special attention should be given to the working time (pot-life) of such mixes since high amounts of GGBFS could lead to flash settings. The fast hardening of geopolymers dramatically affects field work by limiting the transportation opportunity window (plant to site).

### 2.2. Activators/Hardeners

To successfully produce a geopolymer network, in addition to precursors, an alkaline solution is essential for bonding aggregates and materials together through a specific chemical reaction. Moreover, despite their lower popularity compared to alkaline solutions, an acidic medium (e.g., phosphate) could also be used for geopolymer production [16,61]. The typical alkaline solution includes sodium or potassium hydroxide and soluble silicates. The hydroxide helps with the dissolution of the aluminosilicates available in the mixture and the soluble silicates act as the main binding source in geopolymer mixtures [62]. Based on the literature, different activator-related variables could affect the final properties of geopolymer cements and binders. These parameters include alkali cation type (Na^+^, K^+^), soluble silicate alkalinity, the molarity of the activators, and hydroxide to silicate ratio. In addition, the solid to liquid ratio, Si/Al and (Na^+^, K^+^)/Al ratio also influence mixtures’ final properties [63,64,65].

The most common alkali cations used during geopolymer preparation are sodium and potassium. It is indicated by the literature that Na^+^ cations are better in regards of promoting aluminosilicates than K^+^ cations [16]. The ease of handling or workability is an important factor in geopolymer applications, which is determined by molecular ratio, density, and temperature of soluble silicates. For instance, the viscosity of Na^+^ silicates increase with an increase in molecular ratio. However, potassium-based alkaline solutions are much less viscous compared to the Na^+^ hydroxide solutions. Moreover, despite it being less effective for the dissolution process, potassium-based activators could achieve higher compressive strength value compared to sodium-based activators. Higher compressive strength and lower viscosity of potassium mixtures compared to sodium solutions are observable in literature [22,37]. For instance, it was indicated that the compressive strength of samples made with fly ash and loess increased from 29.5 to 113.8 MPa when KOH was used instead of NaOH as the activator. [22].

The soluble silicates act as binders for the geopolymer matrix and its history dates to 1640 where it was introduced by Van Helmont. However, the term waterglass was first coined by Johann Nepomuk Von Fuchs in 1818 when he mixed silica with caustic soda potash (i.e., potassium compounds and potassium-bearing materials). The waterglass was used as glue, cement, paints, detergents, and hardening materials in artificial stone production [16]. Currently, the soluble silicates are used as detergents, chemical applications, and as adhesives. Various studies have evaluated the effect of sodium or potassium silicates on geopolymer mixtures. For instance, it was claimed that its addition to the solution could approximately double the compressive strength of the fly ash-based geopolymer samples [49,66]. The soluble silicates used in different studies vary in terms of molecular weight and chemical structure (Table 2). The molar ratio is also an important factor since it is directly related to the corrosiveness of the solutions. In general, the molar ratio (MR) (SiO_2_:/Na_2_O) < 1.45 is known to be corrosive which may cause problems during handling for the workers. Furthermore, higher MR will lead to longer setting values. A study conducted by Kastiukas et al. (2017) investigated the effects of different SiO_2_-Na_2_O ratios by varying sodium silicate (SS) to sodium hydroxide (SH) ratios [67]. The authors suggested 3.6% for Na_2_O concentration when proportioning SS/SH. In addition, the study used up to 40% of waste glass, which led to a reduction of 22.5% in sodium silicate usage. In a different study, Manjarrez et al. (2019), varied the ratio of sodium silicate to sodium hydroxide from 0.0 to 1.5 in an attempt to understand its effect on compressive strength of geopolymer samples [33]. The maximum strength of 23.5 MPa was observed for 10 M at SS/NaOH = 1.0.

The molar ratio of different soluble silicates is also important in terms of free metal cations and (NA^+^, K^+^)/Al ratios. To have a chemically stable structure, the Na/Al ratio should be equal to 1. Furthermore, the Si/Al ratio depends mainly on the type of geopolymers application. A ratio lower than three is suggested in the literature for geopolymer cement and concrete production [64,68]. In most cases, the ratios are calculated regarding the molar ratios of each element. However, the reactivity of used materials may differ, and the final leaching of Si and Al elements is related to both the chemical composition and mineralogy of precursors. Therefore, a simple method for determining geopolymer reactivity has been proposed by Davidovits [16], where the same amount of activator is used with different amounts of precursors. The hardness of the mixture is measured with a penetrometer after 1 and 2 h. Lower penetration values are an indication of higher mixture reactivity. This method is also appropriate for identifying the flash setting time that could be identified as a mix hardening time less than 30 min. Finally, the minimum amount of required precursors could be determined.

The amount of activator has a great influence on the geopolymer properties considering the solid–liquid ratio. Scanning electron microscopy was used to study the microstructure development in geopolymer pastes. It was concluded that a solid to liquid ratio of 4 showed higher mechanical strength, cohesiveness, and lower micro-crack and micropores compared to lower ratios [37]. Water makes the movement of the particles within the geopolymer gel possible, where the chemical reactions take place. In each reaction cycle, part of the water is reintroduced into the mixture. Thus, it is essential to prevent any water (evaporation) during mixing and curing stages [69]. High liquid to solid ratios could show adverse effects on the final compressive strength of geopolymer samples. For instance, the strength of vanadium geopolymer samples reduced from 23.89 to 5.23 MPa when water to binder ratio was increased from 0.28 to 0.44 in the mixture [42]. Excessive addition of water could decrease the alkalinity of the liquid hardeners and therefore, less dissolution of Si and Al element will occur. Consequently, the compressive strength will reduce significantly [62].

## 3. Quarry Dust and Mine Tailings as Precursors

Bedrocks and unconsolidated deposits are the main sources for most of the obtained aggregates worldwide. In this regard, most of the mineral aggregates are collected from surface-mined stone quarries or from sand and gravel pits. The first step in every quarrying process starts with the stripping stage [70]. This step is critical for improving the overall quality of the products since the unwanted material will be removed from the mining surface minimizing the variations of materials passing n. 200 sieve size. The mineral fillers either obtained through quarry blasting or from excavating gravel deposits are then prepared for the crushing stage. The aggregates go through a stage called scalping which is primarily used for diverting and separating fines from coarse aggregates during the first crushing stage. This is very important since the fines can be removed from the production process if the specifications are not met. The unwanted materials can be piled as waste or used as lower quality aggregates. Depending on the type of rock being processed, the material can undergo up to three crushing stages, which usually uses jaw or gyratory crusher. The crushed aggregates usually have long and thin shapes that are not suitable for asphalt pavement production. Consequently, the impact crushers are preferable. The materials are then gathered in a surge pile and stocked for further processing as required. The second and third crushing stages usually use cone and roll crushers. At the final step, the crushed aggregates go through the screening stage which is vital for quality and gradation control of the materials. Annually, aggregate production process produces huge amounts of by-products which are referred to as mine tailing, quarry dust, or waste mineral fillers by various researchers. These by-products can consist of water, heavy metals, and toxic substances depending on their origin [71]. Different methods have been undertaken for recycling quarry and mine tailings. As for one approach, the production of geopolymer cement and binder using quarry waste has gained attention. Various types of tailings from quarries and mines have been studied which include silt, tungsten, vanadium, iron, gold, copper, zinc, granite, marble, lithium, and phosphate. In this section, the production of geopolymers using different mine tailings IS summarized.

### 3.1. Silt

Lampris et al. [72] collected waste silt from five different aggregate washing plants in the United Kingdom for the production of artificial aggregates through geopolymerization. The geopolymers were constructed using silt and mixes containing metakaolin or fly ash. The latter showed a higher compressive strength of about 30.5 MPa which is more than the mechanical properties generally required for aggregates for building materials. It must be mentioned that the varying chemical composition of waste silt can adversely affect the final quality of the products. This paper is one of the very few which have investigated the recycling of waste silt into geopolymer applications. In one of the most recent attempts, Coode Island silt was improved using fly ash and slag with different proportions of sodium and potassium liquid activators [73]. The authors suggested that the resulting geopolymers could be appropriate for deep soil mixing projects. Interestingly, silt obtained from the Yellow River was used in a geopolymer binder for the production of an artificial flood-preventing stone [74]. It was claimed that the addition of slag had dramatically increased the final compressive strength of the samples. Loess particles which include silt and clay were utilized in a fly ash geopolymer [22]. The loess samples were stabilized successfully using different alkaline activator types including sodium and potassium hydroxides. In a similar work, a case study was conducted in a reservoir located in southern Italy [75]. The aim of the paper was to deal with the loss of water storage capacity of the lakes through alkaline activation of silt residues. The results indicated that the clay and silt could be calcined and reused in applications such as binder, precast, and bricks through alkaline activation.

### 3.2. Tungsten Ore

**Figure 2:**
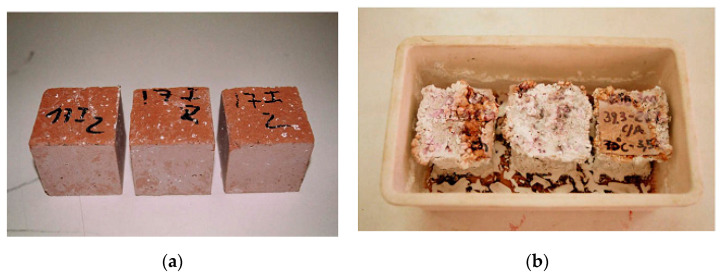
(**a**) mine waste calcinated at 950 and (**b**) mine waste calcinated with sodium carbonate after water immersion, reproduced with permission from [76].

Tungsten was one of the most popular mine tailings waste investigated during the past decade, where different studies have tried to increase its reactivity by means of different methods. For instance, calcination of mud waste obtained from tungsten mining process with sodium carbonate was investigated by Pacheco-Torgal et al. (2010) [76]. The authors observed high efflorescence of sodium after water immersion (Figure 2). Despite achieving high strength values, this method did not achieve favorable thermal reactivity rates. The activation of tungsten mining waste was also investigated through the addition of waste glass [30]. For instance, the addition of 40% of waste glass provided additional silica in the geopolymeric mixture and thus improved the overall strength of the samples by 127% compared to samples containing zero percent of waste glass [67]. Higher silica rates produced 3D structural networks (Si/Al of 2:1).

The durability of the geopolymer has also been tested against acid resistance, where higher acid and abrasion resistance for samples made with tungsten mine waste compared to ordinary Portland cement was observed [77,78]. In a different approach, tungsten mine waste has been used in grouted pavements [79]. The samples made with geopolymer grout presented the lowest compressive strength compared to typical mixtures. It was also concluded that the curing conditions of the mixtures containing geopolymers required further evaluation. Tungsten ore and waste has also been used in different types of pavement applications. For instance, Sangiorgi et al. [26] investigated different recycling scenarios of the waste aggregates and powders obtained from the Panasqueira mine located in Portugal. Additionally, the economic and social impact of the potential recycling techniques were estimated for the local communities. The authors recommended various methods for recycling the tailings of the Panasqueira mine such as its incorporation in the road construction field. However, the focus of the study was to produce alkali-activated composites from mining and quarrying waste based on the available and demanding requirements of authorities. It was suggested that the production of such material could be a competitive and viable solution, which could lead to the manufacturing of aggregates for road paving materials. Similar studies related to the same project verified that artificial aggregates can be produced through the geopomymerization of tungsten mine tailings [38,80].

### 3.3. Vanadium Ore

**Figure 3:**
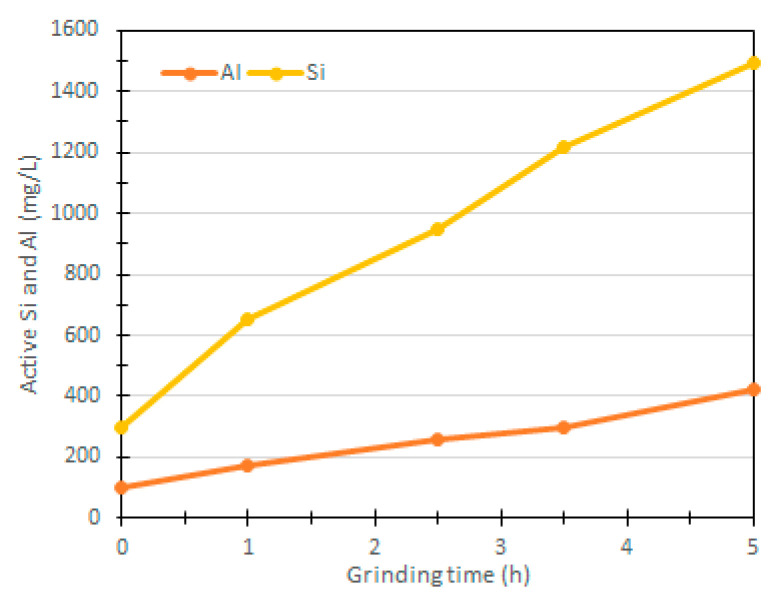
Mechanical activation of vanadium mine tailing, reproduced with permission from [81].

The demand for vanadium extraction has risen significantly and therefore, the mine tailings could turn to possible threats for the environment. The papers reviewed have used vanadium tailings as precursors in geopolymer production. Different methods have been used to reactivate the inert tailing mines such as mechanical activation. For instance, planetary mill ball was used to crush vanadium tailings to smaller particles for a maximum duration of 5.5 h [81]. As shown in Figure 3, an increase in milling time increased Si and Al elements during leaching tests. The authors concluded that the mechanical activation had destroyed the crystalline structure of the material, which led to higher compressive time and faster setting duration.

A different approach was considered to increase the reactivity of vanadium mine tailings. In a study conducted by Jiao et al. (2011), solid NaOH was roasted with the vanadium tailing for 1 h at 450 °C. After cooling down, the mixture was mixed with metakaolin and distilled water. The highest compressive strength was obtained with the addition of 30% metakaolin and curing temperature of 60 °C [32].

### 3.4. Marble

**Figure 4:**
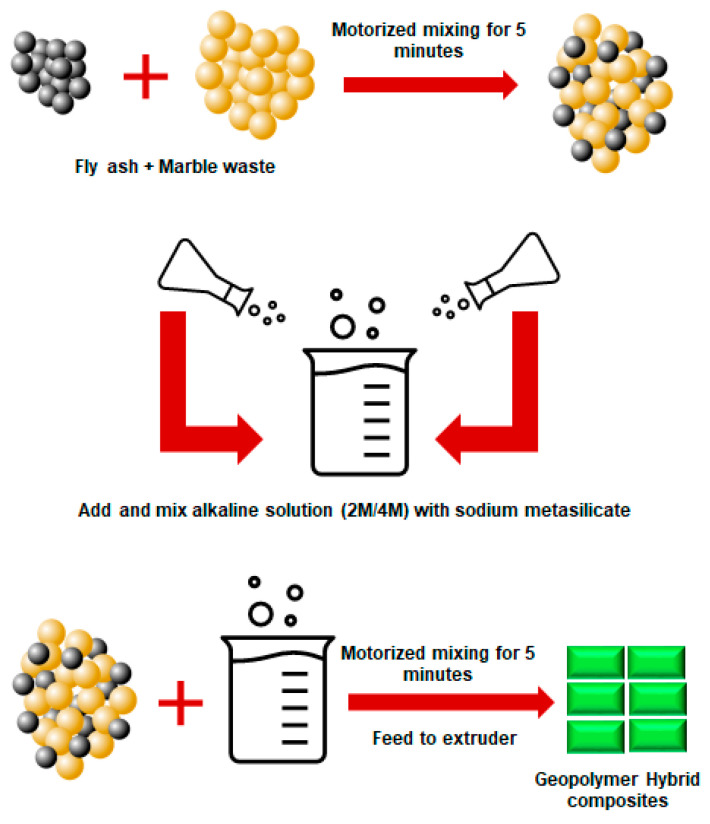
Mixing procedure for marble-based geopolymer cement, reproduced with permission from [82].

During quarrying and different processing methods, huge amounts of marble waste are produced worldwide. Mable stone is the result of metamorphism of sedimentary carbonate rocks such as limestone or dolomite rocks. Like other mine tailings and quarry dust, studies have considered using marble stone waste as precursors in geopolymer production. In a study from Thakur et al. (2019), different molarities of NaOH (2 and 4 M) and sodium metasilicate were used in the production of fly ash-based geopolymer containing marble stone waste [82]. Marble waste and fly ash were initially mixed prior to the addition of the alkaline solution. The mixture was mixed and molded into rectangular casts (Figure 4). The highest compressive strength was related to the mixtures made with 4 M NaOH. The authors highlighted that both chemical properties and mineralogy of the precursors used (fly ash and marble waste) are of paramount importance during geopolymer manufacturing. The amount of reactive Si and Al leached during the chemical reactions may differ from the values obtained during XRF analysis. Therefore, regardless of geopolymer precursor type, it was recommended that the mixing procedure of geopolymers should begin with the preparation of geopolymer paste (e.g., fly ash and liquid hardener/activator). The next step should be the addition of less active precursors or fillers such as marble waste. Otherwise, the silicate present in the activators will not fully engage in the chemical reaction and will remain inside the mixture. This will result in glossy surfaces after the curing process since the unreacted silicates will move to the surface of the sample [16].

### 3.5. Iron Ore

**Figure 5:**
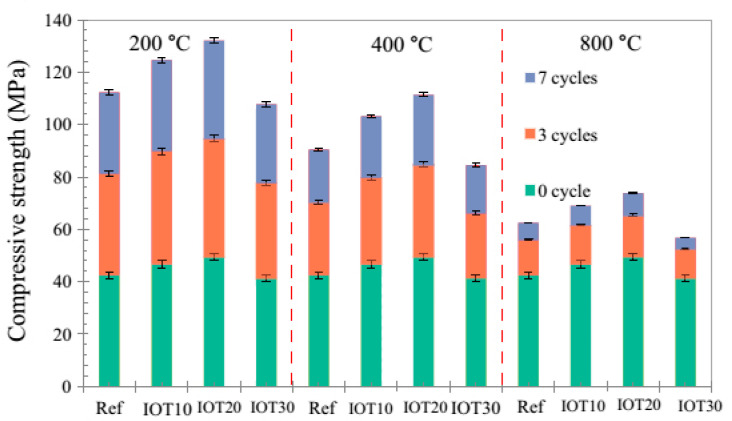
Compressive strength of iron ore tailing based geopolymers exposed to different heat cycles, reproduced with permission from [86].

Iron ore tailings are produced in vast amounts during iron extraction and mining process. Studies have proposed different methods for incorporating mine tailings into geopolymer binders and cements. For instance, the application of iron ore tailing as fine aggregates into mortar made with fly ash was investigated. The maximum compressive strength obtained was 8.27 MPa after 28 days of curing [83]. The production of geopolymer bricks using iron ore tailings was also investigated by different researchers [35,84]. One of the major advantages of producing geopolymer bricks is the avoidance of using high-temperature kiln firing used during traditional production procedures. For instance, geopolymer bricks achieved high compressive strength of 50.53 MPa when cured at 80 °C for 7 days [84]. The authors stated that the produced bricks met ASTM and Australian standard requirements set for bricks. In addition to geopolymer bricks, the possibility of producing lightweight aggregates using iron ore and foam-gel casting technique was investigated [85]. The behavior of iron ore tailing geopolymer samples exposed to different heat-cooling cycles of up to 800 °C was monitored [86]. The samples were made by replacing 10, 20, and 30% (IOT10, IOT20, IOT30) of the fly ash with iron ore mine tailings. The authors stated that the addition of more than 20% of iron ore tailing could significantly increase the setting time of the geopolymer cement. In addition, the loss of compressive strength at different thermal cycles were lower in sample made with iron ore tailings compared to those of the control samples (Figure 5).

### 3.6. Gold and Copper Ore

The immobilization of heavy metals is one of the major priorities in gold mine waste management. Because of its effective capability, geopolymers are widely used as a tool to reduce the impact of heavy metal leaching on the environment. Therefore, various studies have used geopolymers to encapsulate gold mine wastes [53,56,87]. A recent study evaluated the possibility of using gold mine tailing obtained from Finland as a precursor [58]. The Si/Al and the Na/Al ratios ranged between 2.5–3.5 and 0.8–1.5, respectively. The authors investigated leaching values after 7, and 28 days and 18 months. The geopolymer leaching values for the most problematic elements including As, Sb, B, and V were reported to be very low after 18 months of curing. The addition of slag to gold mine tailings allowed for room temperature curing which is in accordance with similar studies [53]. The studies on heavy metal leaching have been followed up by applying a response surface method to better understand the properties of samples made with gold tailings [88].

Copper mine tailings have been used in different applications. Ahmari et al. (2012), mixed NaOH with copper tailings [89]. The aim was to produce suitable alkaline activated material to be used as base layer in pavement construction. The results indicated that the correct amount of NaOH could increase the unconfined compressive strength of the samples compared to the reference material. The findings were similar to a work conducted by Manjarrez et al. (2018) [46]. It was suggested that using appropriate moisture content and NaOH concentration could produce geopolymers, which could satisfy the strength requirements for cement treated-base specified by various road agencies in the United States. The potential of using copper tailings in different applications was examined through different literature. Cement kiln dust (CKD) was added to bricks made with NaOH and copper tailings [90]. It was shown that the addition of CKD significantly improved the durability and mechanical properties of the mixtures. Another application of copper tailing was to produce lightweight aggregates where it was used in cement and mortar mixtures. Compared to LECAs, the lightweight aggregates showed higher mechanical properties and density [52]. The Si/Al and Na/Al ratio of copper waste geopolymers were enhanced by the addition of aluminum sludge [91]. As expected, the addition of aluminum and optimum amount of NaOH significantly increased the compressive strength of the samples.

### 3.7. Phosphate, Lithium, and Basalt

**Figure 6:**
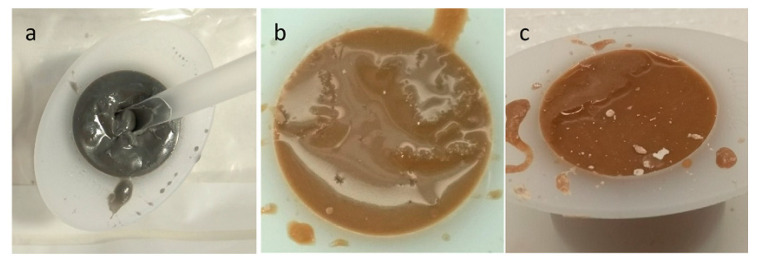
Lithium geopolymer calcination at different temperatures (**a**) not calcined, (**b**) calcined at 750 °C and (**c**) calcined at 900 °C, reproduced with permission from [92].

Most of the tailing mines may be rich in Si and Al elements. However, the reactivity of the material used as precursors is also an essential parameter. One method of increasing the reactivity of the precursors is to calcine the mine tailings. For instance, the effect of thermal treatment on phosphate, kaolinite, and lithium mine tailings was investigated [92]. Regarding lithium tailings, even calcination did not result in a proper dissolution of material and therefore, the authors were not able to examine the mechanical properties (Figure 6).

On the other hand, the best results were observed at calcination temperature of 700 °C for both kaolinite and phosphate mine tailings [92]. Currently, efforts are being made to better understand and evaluate the most determinant and critical parameter that affects the mechanical and chemical properties of geopolymers. For example, statistical analysis is being more frequently applied to aid the identification of important parameters. In this line, the results of the response surface method study indicated that curing temperature was the most critical factor related to the strength of material [93].

Basalt is an igneous rock widely available in the earth’s crust, where its high recyclability and low impurities have allowed it to be used in various applications [94]. Because of its high alumina and silica content, geopolymers containing basalt precursors have become an interesting topic for various researchers. However, in most cases, the focus has been on basalt fibers and less attention has been paid to basalt powders. It is obvious that the using basalt waste would be much more cost-effective than using raw and virgin material. Waste basalt powder has been used for producing geopolymer bricks and artificial lightweight aggregates. Different ratios of sodium hydroxide and silicates were mixed with MK and basalt powder [95]. A liquid to solid ratio of 0.75 and curing of 24 h at 70 °C achieved the highest compressive strength of 64 MPa. Regardless of precursor type and amount, the activators have either contained sodium hydroxide and sodium silicate or they have only contained one type of hardener. For instance, other studies were conducted on geopolymer-containing basalt and only sodium hydroxide was used as an activator at different activator/precursor ratio from 0.1 to 0.5 [96]. In addition, basalt was obtained as raw material and was milled to a very fine powder. Curing conditions similar to the previous works were applied. The Si/Al ratio of the samples were fixed at 2.8, which produced the maximum compressive strength of 57 MPa. Comparing the results from the two studies suggests that the reduction in mechanical properties could be related to the difference between the alkaline solutions of the studies, where the latter had only used NaOH for its activator rather than a combination of sodium silicate and NaOH. A separate study investigated the possibility of producing novel low impact porous pavement layers using synthetic aggregates [27]. The aggregates were made of geopolymer paste containing 10 M NaOH, sodium silicate, waste basalt powder, and metakaolin. The curing time and temperature were 12 h at 60 °C, which returned the highest compressive strength of 65 MPa.

## 4. Precursor Pre-Treatment and Calcination

**Figure 7:**
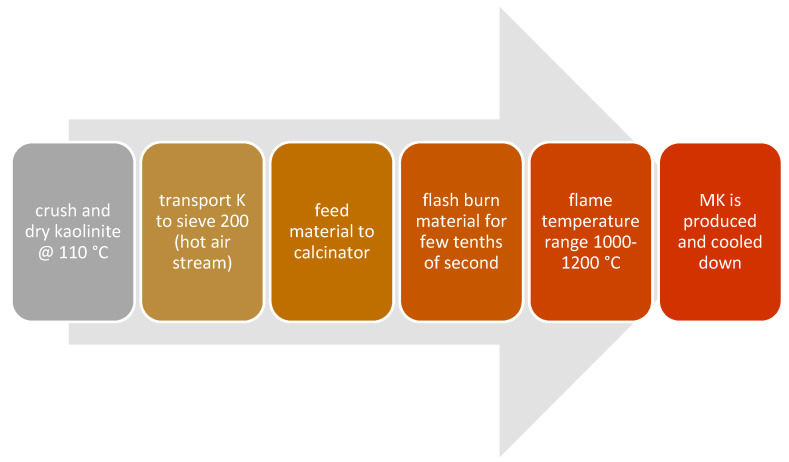
Flash calcination of MK, adapted from [100].

Because of the crystalline nature of various tailings, calcination or thermal treatments have been widely used as a step toward achieving high-performance geopolymer cements and binders [76,77,92,97,98,99]. Three major calcination methods are used at industrial stage which include rotary furnace, multiple earth furnace and flash calcination plants. The methodology behind each method is different. However, the flash calcination plants have gained favorable attention due to their efficiency and relatively lower energy consumption. The overall process of the flash calcination method for MK production is illustrated in Figure 7.

**Figure 8:**
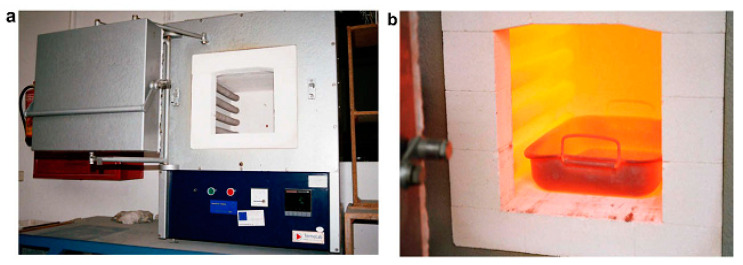
Calcination method for tungsten mine tailing, (**a**) furnace and (**b**) quenching of material, reproduced with permission from [76].

However, the calcination process at the laboratory stage is mostly done with the aid of static furnaces, which can normally heat materials up to 1200 °C. In some cases, the heating rate during calcination is reported. Therefore, at least three important parameters could affect the laboratory calcination of materials, which include the temperature of calcination, heating rate, and calcination duration. It has to be noted that over calcination could decrease the reactivity of the materials due to the formation of inert crystalline structure. Fast cooling of calcined material is suggested since it could avoid crystallization of the raw material during the thermal treatment. It was for this reason that the calcination of mining waste was quenched in water after being exposed to high temperatures for a certain amount of time (Figure 8) [76]. In most calcination procedures, small details could help with the reproducibility of the test for future studies. For instance, one method is to put the samples inside the furnace and then start the calcination process. In this case, a certain time is required to pass for the furnace to reach the target temperature. Then the sample is calcined for a desired duration. Finally, the sample will remain inside to reach room temperatures. However, if the sample is placed in the oven only when the target temperature is reached and is immediately taken out after the desired calcination period has finished, the duration for which the sample is calcined would be significantly different compared to the first method [47].

The production of geopolymers containing clay and silt is highly dependent on the reactivity of the clayey material in question. Therefore, various researchers have attempted to calcine the clay sediments at different times and temperatures to achieve durable geopolymer cement [99,100,101,102,103]. An increase of calcination temperature from 400 to 750 °C resulted in higher leaching of Si and Al. Consequently, enhanced mechanical properties were observed [99]. In most cases, the effective calcination temperature ranged from 600 to 800 °C.

The calcination is not only limited to the precursors. In some cases, attempts have been made to calcine the solid part of activators such as NaOH with different precursors such as copper [104], gold [88], phosphate [61], quarry stone dust [40], and vanadium [32,105]. This method is referred to as alkaline fusion where the NaOH pellets are added to the precursors at different weight ratios. Usually, liquid sodium silicate or water is then added to the mixture to start the geopolymer reaction. The fusion temperature for the reviewed papers ranged from 450 to 800 °C for a maximum of 2 h.

## 5. Curing Conditions

**Figure 9:**
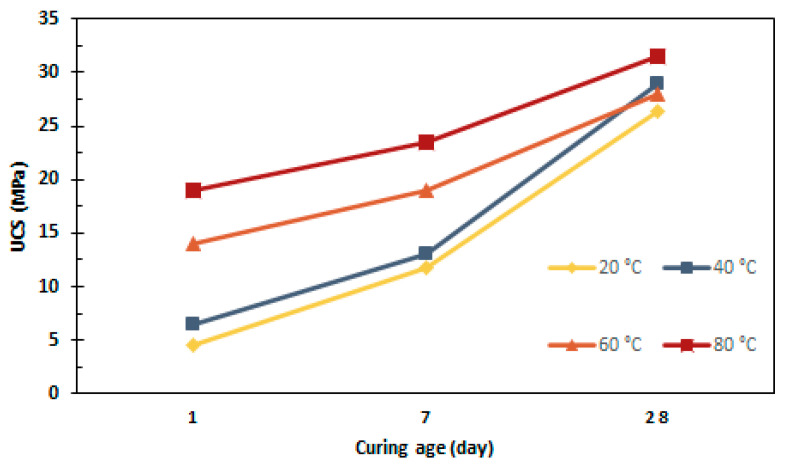
Effect of curing days and temperature on the compressive strength of vanadium mine tailing based geopolymers [42].

Heat curing for an appropriate time and temperature is highly suggested for geopolymers containing low-calcium content. The heat treatment aids the chemical reaction occurring in geopolymer paste and increases geopolymerization process. For instance, the durability of geopolymer mixtures produced with calcinated tungsten waste mud were evaluated at different curing conditions. In the first stage, the samples were either cured at room or elevated temperature of 130 °C between 7 and 28 days. The samples were then placed in water baths for different durations ranging from 0 to 91 days. The study revealed that all samples disintegrated when introduced to water. However, samples cured at 130 °C or cured for longer period showed higher durability. In addition, a significant loss in strength was observed within the first 4 week of water immersion, where the UCS decreased to 1–3 MPa. The low durability in all cases were possibly related to an incomplete geopolymer process/reaction. Moreover, the reactivity of the materials in use may not have been efficient for geopolymer production. Lastly, it was suggested that higher curing temperatures could speed up the initial reactions in geopolymer network, since samples cured at 130 °C showed better durability than room temperature-cured samples [106]. Curing can be achieved through a steam or dry heating system. However, it was noted that the dry curing method showed 15% higher compressive strength than that of steam-cured geopolymer concrete [107]. Figure 9 demonstrates the effect of both curing days and temperature on the compressive strength of vanadium-based geopolymers [42]. The samples were cured in an oven for 24 h. Higher curing time and temperature resulted in higher compressive strength.

**Figure 10:**
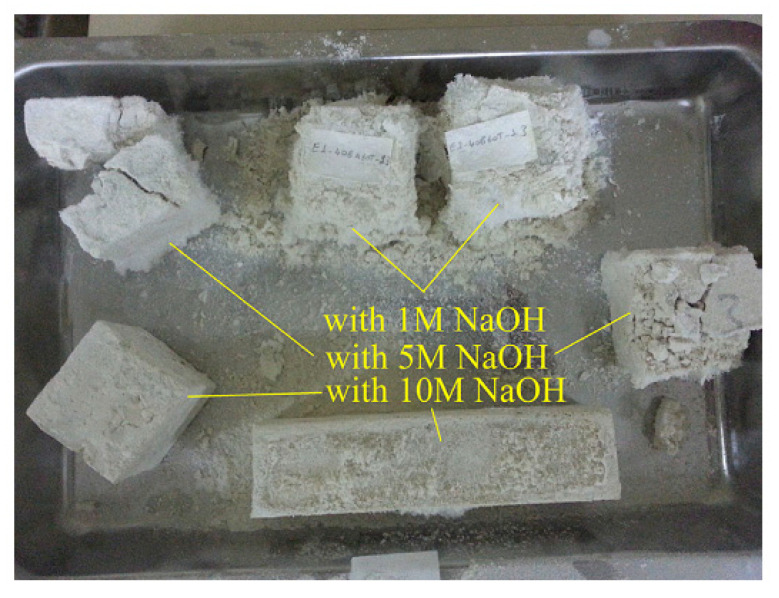
Wet-curing of marble geopolymer cement, reproduced with permission from [109].

Different curing times and temperatures have been used in various studies. For instance, curing was used prior to and after the demolding of geopolymer samples. The geopolymer paste was prepared and cured in an oven for 40 or 60 °C for 24 h. The de-molded samples were then subjected to the same curing temperature for a duration of 7, 14, and 28 days. It was indicated that higher curing temperature and duration resulted in higher compressive strength of the material, where the highest UCS of 14.78 and 20.49 were reported for samples cured for 28 days at 40 and 60 °C, respectively [108]. In some cases, the effect of different curing duration was observed. For example, the curing temperature was kept constant and the effect of different curing durations of 1, 3, and 5 h was investigated on the performance of fly ash geopolymers [44]. The authors found the curing temperature of more than three hours challenging for its implementation in a real construction site. A higher curing temperature of 80 °C for 5 h was suggested to have the best effect on geopolymer compressive strength reaching to 19.2 MPa. Curing duration of more than 24 h decreased the overall geopolymer compressive strength [63]. It is evident from the literature that both curing time and temperature could directly affect the mechanical properties of geopolymers. In a different attempt, samples were cured both in water and at room temperature for different durations [109]. The samples underwent three different curing conditions that included curing at room temperature, the curing in an oven at 45 and 75 °C for 24 h. The samples were then de-molded and half of them were cured inside water for 90 days. The authors indicated that temperatures up to 75 °C can increase the hardening process of the samples. However, wet curing method was excluded because of high efflorescence (Figure 10).

Achieving the highest compressive strength as possible could be the main goal for most studies and projects. However, the sustainability and cost-efficiency of any proposed approach should also be taken into consideration. Based on the literature, it was indicated that temperature and curing conditions could increase the ultimate strength of the geopolymers. Unfortunately, higher temperatures and longer curing conditions consume much more fuel and energy compared to those samples which are cured at room temperature. Consequently, the overall cost of projects and research applying these methodologies will inevitably increase. In addition, the implementation of different curing conditions may not be applicable on a real scale in most project sites.

## 6. Mechanical and Chemical Properties

Engineering properties such as compressive strength, density, durability and acid resistance, water absorption, and abrasion of geopolymers containing mining waste were investigated by different studies. Unconfined compressive strength (UCS) is the most common mechanical property that has been investigated in the literature. The impact of different parameters involved in geopolymer production on UCS has been reported by various authors. This includes the effect of precursor type and ratio [30,33,44,45,82,110], calcination [76,99,100,102,103], activator blends [22,31,63,75,111], liquid to solid ratio [36,37,42,112], Si/Al and Na/Al [91,113,114], and curing conditions [74,106,115]. For geopolymerization, Si/Al lower than 3 is suggested since higher ratios would create linear structures rather than a 3D structure. In addition, one of the most important methods of controlling the leaching of geopolymers is to have a chemically stable structure. This is achieved by reaching a Na/Al of 1 in the geopolymer mix.

Different types of mine tailings waste can provide unique physical strength and properties to geopolymers when combined with the common precursors such as metakaolin, blast furnace slag, and fly ash. Studies on geopolymer produced with copper highlighted that the compressive strength of the samples varied significantly when metakaolin was added as a precursor in the mix design. The maximum compressive strength of 23 MPa was obtained substituting 38% of the total weight with MK. With similar curing conditions, the compressive strength dropped to 18 MPa when the exact amount of MK (38%) was replaced with the blast furnace slag [43]. However, in a separate study, only copper tailings were used [108]. In this case, the highest achieved strength was reported to be 15.70 MPa for samples cured at 60 °C. The difference between the compressive strength of the mentioned studies could be related to the presence of MK and the difference in activator content. Only sodium silicate with different concentrations was used by Falah et al. [108], whereas Paiva et al. [43] used a combination of sodium silicate and 10 M sodium hydroxide as activators. Similar activator content of SS and 10 M NaOH was used for producing lightweight aggregates and paving blocks using basalt powders and MK as precursors [27,95]. With very similar curing conditions, only basalt was activated using NaOH for the production of microcrystalline particles [96]. Samples that benefitted from MK and a combination of both SS and SH [27,95] presented higher compressive strength.

**Figure 11:**
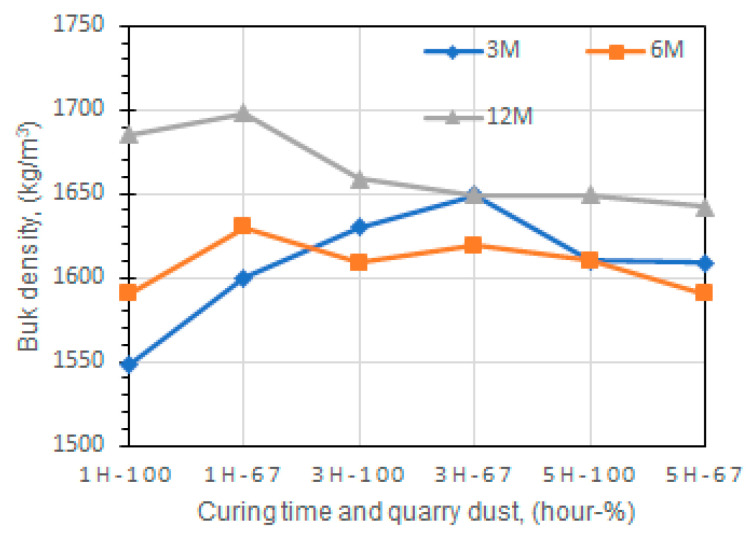
Bulk density-curing time waste glass-based geopolymers, reproduced with permission from [44].

The density of geopolymer made with quarry dust and waste glass was investigated [116], where the addition of geopolymer cement to the soil was able to increase the maximum dry density of the final mixture. In a separate work on the bulk density of fly ash geopolymer [44], the highest obtained value was related to samples made with NaOH (12 M) and sodium silicate, which were cured for 1 h (Figure 11).

Durability is an important aspect of geopolymer science, which has been studied by various researchers [80,84,106,117,118]. One method of studying the durability of geopolymers is for example to immerse the samples in water for a period of 24 h. Doing so would give an overall view of the effect of water immersion on the physical properties of the sample. It was understood that most of the partially alkali-activated specimens were disintegrated after being submerged in water for a period between 0 and 90 days [106]. The key to obtaining a geopolymer structure that would behave similarly to ordinary Portland cement during wet curing conditions is to achieve a chemically stable state. This is obtained by satisfying the required Si/Al and Na/Al ratios [16]. The durability of geopolymers in terms of resistance against sulfide and acidic attacks was also covered by the reviewed literature [77,78]. In this regard, 50 mm cube samples were cured in sulfuric, hydrochloric, and nitric acid solutions for 28 days [78]. The results indicated that both acid and precursor type affected the overall outcome. Acid attacks are observable as weight loss of samples.

Among the reviewed context, less attention was paid to the abrasion and thermal resistance properties of geopolymer samples. The abrasion for geopolymer samples was assessed by calculation of weight loss of samples subjected to 1000 cycles in the Los Angeles Machine [77]. The obtained data indicated higher abrasion resistance of geopolymer samples made with tungsten mining waste than those of ordinary Portland cement. Regarding the resistance of geopolymer specimens against intense thermal cycles, it was shown that the compressive strength of all samples was significantly dropped [86]. However, the experimental data showed that the addition of iron ore tailing had increased the thermal resistance of the samples compared to the sample made with fly ash.

It is noteworthy, that every mine tailing has its own unique mineralogy and reactivity rate, which differs from source to source and type to type. XRF (X-ray fluorescence) and X-ray powder diffraction (XRD) has been frequently used by various studies to determine chemical oxidation and mineralogy (crystalline phase) of geopolymers, respectively. The obtained data are of paramount importance since they could indicate Si/Al and Na/Al of the final geopolymer mixture. For instance, XRD analysis for tungsten mud waste showed that the crystalline phase (quartz and muscovite) was not changed after the polycondensation process [106]. Furthermore, the XRF analysis indicated a decrease of Na_2_O concentrations for samples submerged in water (7–91 days). Since Na^+^ cations did not fully react within the geopolymer network and the excess NaOH activator was dissolved in water (Na/Al ≠ 1) and because of the presence of crystalline phase after the addition of alkaline solution, the authors suggested that the geopolymer reaction was not fully completed. The outcome was excessive leachate of Na^+^ cations freely interacting with water.

The addition of 20% iron ore tailing to fly ash-based geopolymer decreased the porosity and microcracking of samples [86]. Thus, the denser microstructure provided better thermal resistance compared to samples with no iron ore tailings. Scanning electron microscope (SEM) was used to evaluate the microstructure of tungsten mining waste-based hybrid alkaline materials [112]. The optimum mixture containing 10 M sodium silicate and potassium hydroxide as alkaline solution showed a maximum UCS of 29.2 MPa. The compact structure showed the presence of gehlenite hydrates and small cracks were observed because of rapid synthesis of the binder. The outcome suggested that the microstructure of the mixture could be influenced by the molarity and type of activators used for geopolymer production. Adesanya (2020) investigated the effects of mechanical activation on phyllite’s chemical and microstructure properties [119]. The mechanical milling was conducted for 9 and 15 min. The XRD analysis indicated a continuous breakdown of the crystalline phase related to muscovite and chamosite. However, Albite and quartz had no significant change in terms of their crystalline structure after the milling process. It was noted that the amorphous phase had increased by 47% wt. The study concluded that the mechanical activation had led to higher solubility of Si and Al in alkaline solutions.

## 7. Conclusions

The applications of various quarry and mine waste for geopolymer production in the past ten years have been reviewed. The extracted data including authors, publication year, type of precursors, activators, activator to precursor ratio, curing conditions, Si/Al, (Na, K)/Al, UCS, and the mineralogy of the mine tailings are presented in Table 3. The following conclusions could be drawn from the reviewed literature:

- The type of precursor has a direct impact on various mechanical and chemical properties of geopolymers. Inert waste dust could act as a filler/aggregate in the geopolymer structure, whereas reactive quarry waste could act as a precursor in the geopolymer matrix. Therefore, the mineralogy of the precursor being used must be studied.
- The mineral waste was used as a precursor to produce geopolymers. In addition, some studies used the waste-based geopolymers to create artificial aggregates. In both cases, promising results were observed.
- The addition of Mk, GGBFS, and fly ash to quarry/mine waste could improve the geopolymer network by satisfying Si/Al and Na/Al ratios.
- Thermal treatment of mineral waste has proven to increase the amorphous phase and reactivity of the material providing better dissolution of Si and Al in alkali mediums. For instance, some silt and clay materials are rich in quartz and have less reactivity due to their crystalline structure. Calcination could modify the structure and provide a better source of reactive material.
- Based on the literature, different activator-related variables could affect the final properties of geopolymer cements and binders. These parameters include alkali cation type (Na+, K+), soluble silicate alkalinity, the molarity of the activators and hydroxide to silicate ratio. In addition, the solid to liquid ratio, Si/Al and (Na+, K+)/Al ratio also influence the final mixture properties.
- Potassium silicates are less viscous and provide higher compressive strength compared to the more common sodium silicates.
- Curing samples at elevated temperatures increases the reaction rate of geopolymers. Ambient temperature curing has also been practiced in different studies, which have shown acceptable outcomes. Room curing temperature could be aided by including GGBFS in the mixture.
- Water-curing of geopolymer samples has shown adverse effect on the mechanical properties and durability of produced samples. Backed up by various studies, excessive leaching has been related to incomplete polycondensation of the geopolymer network. Excess alkali solution interacts with water and appear as white powder on the surface of products.
- The microstructure of geopolymers could be affected by molarity and type of alkali solution. In addition, mechanical and thermal treatments have shown to change porosity and chemical structure of geopolymers.
- The following recommendations have been proposed:
- A standard method for geopolymer production including step-by-step procedures on selecting proper precursors could be proposed. Geopolymer specific standards for leachate tests as well as in-depth chemical analysis (NRM, FTIR, and XRD) could be included.
- Since various parameters affect the overall performance of geopolymers, acquiring a full understanding of all the involved parameters is very difficult. In this regard, applying statistical methods such as design of experiments (DOE) could reduce the overall time required for understanding the relations between various factors. Moreover, statistical analysis could also provide mathematical modeling that could fine-tune geopolymer mix design and enhance the final performance.
- Using ambient temperature curing systems will facilitate industrial implementations. Most of the proposed mix designs for geopolymers can only be conducted at laboratory scale and industrial aspects have not been foreseen. For instance, heat curing is being widely used in almost every study related to geopolymers. However, curing every produced geopolymer sample at manufacturing plants would dramatically increase the final production costs of geopolymers.
- Considering the cost of metakaolin and alkaline solutions, every effort should be considered to reduce the final cost of geopolymers. Thus, it would be more appropriate to select local raw/waste material which will eliminate transportation and material costs giving geopolymers a more competitive market.
- User-friendly alkaline solutions should be used more frequently.
- The mixing procedure should start with creating the binder. The binder consists of reactive precursor such as MK and the hardener/activator. The mixing should then continue with the addition of less reactive materials such as mineral waste and mine tailings.

## Figures and Tables

**Table 1:** Coal composition before and after combustion, adapted from [16].

Coal Minerals	Phase After Combustion Process		
	850 °C	1500 °C	1800 °C
Quartz	Quartz	Cristobalite	Glass
Kaolinite	Metakaolin	Glass + mullite	Glass
Illite	Illite	Glass + mullite	Glass
Pyrite FeS_2_	FeS/FeO	Hematite + glass	Glass
		Magnetite + glass	
Calcite	CaO	Glass	Glass

**Table 2:** Commercial silicates, adapted from [16].

Silicate Type	MR (SiO_2_:Na_2_O)
Sodium orthosilicate Na_4_SiO_4_	0.5
Sodium metasilicate Na_2_SiO_3_	1.0
Sodium disilicate Na_2_Si_2_O_5_	2.0
Sodium polysilicate Na_2_O.3.3SiO_2_	3.3

**Table 3:** Summary of reviewed studies on geopolymer cement and binders containing mine tailings.

Ref.	Year	Precursors	Activators/Mix Design	A/P	Curing	Si/Al, Na, K/Al	UCS MPa	Mineralogy/Chemical Composition
[115]	2018	Antigorite	SiO_2_/Na_2_O = 2.25	0.25	24 h @RT 1, 7, 28 d	N/A	49 15-wet cure	Serpentine, talc, olivne, pyroxenes, amphiboles, magnetite
[27]	2019	Basalt, MK	SS, SH 10 M, SiO_2_/Na_2_O = 1.99, SS/SH = 4	0.75	12 h @ 60 °C	N/A	65	N/A
[95]	2019	Basalt, MK	SS, SH 10 M, SiO_2_/Na_2_O = 1.99, SS/SH = 4	0.75	24 h @ 70 °C +3–28 d	N/A	64	N/A
[96]	2017	Basalt	NaOH	0.1–0.5	24 h @ 80 °C	Si/Al = 2.8	57	Plagioclase, clinopyroxene, olivine, and magnetite
[110]	2019	Boron, calcined	Sodium silicate consisted of 8% Na_2_O, 27% SiO_2_, and 65% H_2_Oby mass NaOH	~0.40	Heat treatment cycle	N/A	38	Colemanite, hydroboracite, illite, montmorillonite, calcite, and quartz
[108]	2019	Copper	SiO_2_/Na_2_O = 3.2 6 M	0.23	7, 14, 28 @ 40, 60 °C	10–38 Si/Al 0.45–2.80 Al/Na	6.44–15.70	Tremolite, chlorite, quartz, talc, magnesium silicate hydrate, dolomite, and calcite
[43]	2019	Copper, slag, MK	SS, SH 10 M	0.1 + water	7 d @ 20 °C	Si/Al = 2 Na/Al = 1	4–45	Piryte, anhydrite, caldecahydrite, quartz
[33]	2019	Copper	NaOH 5, 10, 15 M, SS/SH 0, 0.5, 1.0, 1.5	0.158	45–85 °C, 7 d	~3–5 Si/Al	23.5	Quartz, gypsum, and albite
[46]	2018	Copper	NaOH, 0, 3, 5, 7, 11 M	11–19% water	7 d @ 35 °C	Na/Al 0.3–1.1	5.32	Quartz, gypsum, albite, sanidine
[104]	2017	Copper, calcined	NaOH @ 550–650 °C	N/A	N/A	Leaching test	N/A	Quartz, albite, chlorite, dolomite
[52]	2017	Copper, zinc, fly ash, gold	SS, SH, SiO_2_/Na_2_O = 2.5	W/c 57–63	28 d @ RT	N/A	~30	N/A
[57]	2015	Copper, slag	NaOH 15 M	16% water	7 d @ 60, 75, 90, and 105 °C	1.8–3.2 Na/Al	75	Quartz, albite, sanidine, gypsum
[91]	2015	Copper, aluminum sludge	SH	0.8–1.3	7 d @ 90 °C	0.08–6.75 Si/Al 0.34–1.3 Na/Al	~45	Quartz, albite
[90]	2013	Copper, ckd	NaOH 15 M	12–20%	7 d @ 90 °C	3–14 Si/Al 0.8–3.0 Na/Al	~50	Albite, gypsum, quartz, sanidine
[117]	2013	Copper	NaOH 15 M	16% water	7 d @ 90 °C	7.76 Si/Al 0.86 Na/Al	~35	Albite, gypsum, quartz, sanidine
[66]	2012	Copper	(SiO_2_/Na_2_O) of 3.22 NaOH 5, 10, 15 M	33% water	60–120 °C 7 d	1.67–8.88 Si/Al 0.96–2.82 Na/Al	N/A	Albite, gypsum, quartz, sanidine
[120]	2012	Copper	NaOH 10, 15	8–18%	60–120 °C 7 d	0.86–1.94 Na/Al	33.7	Albite, gypsum, quartz, sanidine
[89]	2012	Copper	NaOH/copper 0, 2, 4, 6%	15%	7–90 d, RT	N/A	~3	N/A
[71]	2019	Garnet, MK	SiO_2_/Na_2_O = 3.1 NaOH, SS	0.43	20–80 °C 3 d	Garnet has less than 2% Si, Al release	46	Grossular, andradite, dolomite, calcite, quartz, almandine, boehmite
[88]	2019	Gold, Al_2_O_3_	SH+gold @ 550 °C for 1 h +SS	N/A	3 d Temp based on RSM 25–89 °C	14.6 Si/Al	N/A	89.25% SiO_2_, 6.1% Al_2_O_3_, 1.24% K2O, 0.84% Fe2O3, 0.29% mgo, 0.09% SO3
[113]	2019	Gold	NaOH, KOH 2–10 M Good for paper	0.1–0.5	N/A	Leaching tests	N/A	N/A
[121]	2019	Gold	10 M KOH, KS, KA KS:KH 0–2	0.26	5 d @ 80, 100 °C	N/A	18	Silicon dioxide (SiO_2_), magnesium oxide (mgo), aluminum oxide (Al_2_O_3_), potassium oxide (K2O), and ferric oxide (Fe2O3),
[58]	2018	Gold, MK	NaOH 8, 9 M SiO_2_/Na_2_O = 3	N/A	28 d @RT	1.8–2.6 Si/Al Na/Al = 1	N/A	N/A
[53]	2018	Gold, MK, slag	NaOH 8 M, SS/SH = 1.25 SiO_2_/Na_2_O = 3.0	N/A	24 h @ RT 7, 28 d	Leaching test	N/A	N/A
[87]	2018	Gold	Leaching test	-	-	-	-	Quartz, chlorite, magnetite, jarosite, pyrophyllite, albite, clinochlore, sepiolite
[56]	2016	Gold, slag	NaOH 5, 10, 15	0.17–0.25	28 d @RT	N/A	22	Albite, gypsum, quartz, dolomite, pyrite, sodium aluminum silicate
[45]	2019	Iron, wool glass	NaOH 8 M, 10 M, and 12 M Mechanical activation	0.27	7 d @100 °C	4.59 Si/Al	112.8	Chamosite, Chantalite, Quartz, Geothite, Hematite
[83]	2018	Iron, fly ash	SS, SH 10 M, SS/SH = 2.5	0.4–0.8	14, 28 @ RT	N/A	8.27	N/A
[85]	2018	Iron	Silicate cement, NaOH,	0.7–1.0	12 h @RT	N/A	5.18	N/A
[84]	2016	Iron brick picture	SiO_2_/Na_2_O = 3.58	30%	3 d @ 80 °C Combination	N/A	50.35	Quartz, goethite, aluminian, birnessite, and sodian
[36]	2011	Iron, Slag	SS, SH 10 M, SS/SH = 0.5 SiO_2_/Na_2_O = 3.6	0.35	7 d @ 30 °C	N/A	63.79	Quartz
[86]	2016	Iron, fly ash	NaOH 10 M, SiO_2_/Na_2_O 3.2, SS/SH mass ratio = 9.14	0.5	28 d @ RT	N/A	50	Anatase, antigorite, albite, amphibole, calcite, chlorite, dolomite, gypsum, quartz hematite, mullite, muscovite, pyrite
[92]	2019	Lithium, phosphate, mk	SS, SH	4	24 h @ RT	Leaching test	~16	Calcite, phlogopite, muscovite, quartz, kaolinite, albite, microcline
[82]	2019	Marble, fly ash	SS, SH 2,4 M, SS/SH = 1	0.22–0.3	24 h @ 70 °C +7 d	N/A	6.52	Calcite, dolomite, quartz
[51]	2018	Marble, gypsum, fly ash, clay	SS, SH 8 M, SS/SH = 5	N/A	28 d @ RT	N/A	52	N/A
[122]	2017	Marble, slag	SiO_2_/Na_2_O = 0.96–1.40 NaOH, SS	0.4	28 d @ RT	N/A	42–60	N/A
[109]	2016	Marble	NaOH 1, 5, 10 M	~0.5	24 h @ 20, 45, 75 °C +2–90 d	Si/Al > 3	Wet curing	Quartz and zeolite
[61]	2019	Phosphate, MK	(NaOH + phosphate) calcined @550 and 800 °C + SS	0.4	−24 h RT −4 h @ 60 °C −28 d RT	2.4 Si/Al @550 °C 3.61 Si/Al @800 °C	40	34.09% fluoroapatite, 12.44% quartz, 11.15% calcite, 9.45% dolomite, 9.46% illite, 21%, palegorskite, 1.4% hematite
[119]	2020	Phyllite	SS, SH	0.29	−RT, 60 °C −7 and 28 d	0.45 for Si/Al	2–25	Quartz, muscovite, chamosite, albite
[44]	2019	Fly ash, quarry dust	SH 3, 6, 12 M SS/SH = 2	0.72	1, 3, 5 h @ 80 °C	N/A	19.2	Calcite mineral as well as dolomite, carbon and other minerals
[123]	2018	Red mud, mk, RHA	NaOH 5 M, SS/SH = 4	0.97	24 h @ 70 °C 60 d	3.85, 4.30, 4.45 and 5.30 Si/Al	30	Hematite, goethite and sodalite
[111]	2018	Silt, Fly ash, slag	SH, KH, 8 M, SS, KS	1	3–28 d	Leaching test	8	N/A
[103]	2017	Clay, calcined	NaOH 14 M, SiO_2_/Na_2_O = 3.2	0.63–1.88	24 h @ 20, 60 °C 21 d	Na/Al = 0.85 Si/Al 3.64–5.03	22.9	Quartz, calcite, kaolinite, illite, smectite, and albite
[74]	2017	Silt	SH	10% water	Air and wet cure	N/A	~12	Quartz, sodalite, albite, calcite, microcline
[22]	2016	Loess, fly ash	NaOH, KOH,	0.2–0.7 water	RT	Si/Al = 2–3	113.8	Muscovite, nimite, quartz, albite, calcite
[124]	2016	Quartz, clay, MK	KS, KH	0.3–0.34	1, 7, 28 d @ RT	N/A	~20	Quartz
[125]	2015	Clay	SS, SH, KOH	0.5	3–28 d @40, 80 °C	Si/Al = 2	7.58	Montmorillonite, alkali feldspar, and quartz
[102]	2015	Silt, calcined	Sodium and potassium aluminate 8–17 M Combination	N/A	15 min–28 d @ 60 °C	Al/NA = 0.647 Al/K = 0.53	6.7	Kaolinite, smectite, and illite (62% total), quartz (18%), calcite (17%) and feldspars (3%)
[99]	2015	Clay, slag calcined	SH 5, 7, 10 M SS	0.4	3 d @ 60 °C 28 d	Na/Al = 1 Leaching test	1.7–38.90	Quartz (18%), calcite (17%), feldspars (3%), clay phase (62%)
[29]	2013	Silt, fly ash, slag	NaOH	0.28–1.20	3, 7, 28 d @RT	N/A	N/A	Gismondine, quartz, illite, montmod Uonite
[101]	2013	Silt calcined	NaOH 5 M	N/A	3 d @ 60 °C	Na/Al = 1	N/A	Quartz, calcite, kaolinite, illite/smectite
[41]	2019	Sphalerite, MK	1 mol Na2SiO3 and 12 mol H2O +Pb(NO3)2	0.49	−6 h @ 60 °C −RT for 7 d	N/A	2–15.5	45.72% dolo- mite, 35.26% calcite, 5.22% kaolinite, and 3.69% quartz
[40]	2019	Stone dust, fly ash	Stone dust: NaOH 1:1.6 @ 500 °C	N/A	−24 h @ 30 and 80 °C −RT or water curing for 96 h	N/A	N/A	SiO_2_ and caco3
[37]	2020	90% tungsten, 10% slag	SH 8 M, SS, SiO_2_/Na_2_O = 3.23, KOH 8, 10 M, waste glass	1/3, 1/4	-Primary 60 °C for 24 h −7, 14, 28, 56 and 90 d	~0.45 for Si/Al 0.104–0.214 for Na/Si	~17–33.05	Muscovite and quartz
[38]	2019	Tungsten, glass waste	Foaming agents: Al powder, SDBS, mno2 Dosage of Na_2_O (3.1%, 3.3%, and 3.5%) 10 M SH+SS	0.22	−40 °C, 60 °C, 80 °C, and 100 °C −7 d	N/A	3.8	Only chemical composition
[30]	2019	Tungsten, glad, MK	SiO_2_/Na_2_O = 3.2 NaOH 10 M, SS/SH = 3	0.4	24 h @ 60 °C 28 d	N/A	2.28–16.10	N/A
[67]	2017	Tungsten, glass	SiO_2_/Na_2_O = 1.75 NaOH 10 M	0.3	24 h @ 80 °C	3 = Si/Al	61	N/A
[63]	2017	Tungsten	NaOH 10 M, SS/SH = 4	1/3.6	Various combination	N/A	41	Muscovite, silica, sodium aluminosilicate, albite, pyrite
[79]	2016	Tungsten, grout	Na4SiO4 and NaOH 12 M	N/A	7, 14, 28 d, RT	N/A	14.3	N/A
[106]	2012	Tungsten	SS, SH 10 M	0.25	48 h @ RT +water cure 7–28 d	Si/Al = 3–4	~18	Quartz, muscovite
[77]	2010	Tungsten	NaOH 12 M SS/SH = 2.5	1.8%	24 h @ RT	Leaching test	8.4–39.6	Muscovite, quartz
[76]	2010	Tungsten	SH 12 M, SS, sodium carbonate	0.5	7, 14, 28 @RT	N/A	~45	N/A
[42]	2019	Vanadium, MK	NaOH	0.28–0.44	3, 7, 28 d @20–80 °C	N/A	31.5 mpa	Quartz, potassium mica
[39]	2019	Vanadium, MK, calcined	(NaOH + precursor) + water	0.35	7 d @RT	Leaching test	29.0	N/A
[81]	2017	Vanadium, MK	SS	0.36	7, 14 @ RT	Si/Na/Al = 3:2:1	~25	Quartz, feldspar, plaster, hematite
[32]	2011	Vanadium, MK	NaOH @ 450 °C	N/A	12 h @ 20 °C 7 d	Na/Al = 0.8 1.5–2.35 Si/Al	55.7	quartz, feldspar, diopside
[105]	2011	vanadium	NaOH @ 750 °C 2 h SA	N/A	RT 3 d	6 Si/Al 0.8 Na/Al	36.2	N/A
[126]	2019	Zinc, MK	SS	0.49	6 h @60 °C +7 d	Leaching test	30	Quartz, calcite, andradite

A/P: activator to precursor ratio; UCS: unconfined compressive strength; SS/SH: sodium silicate to sodium hydroxide ratio; SS: sodium silicate; SH: sodium hydroxide; KS: potassium silicate; KH: potassium hydroxide; RT: room temperature; MK: metakaolin; M: molar; N/A: not applicable; @: at; d: days.
